# ZIKA virus infection causes persistent chorioretinal lesions

**DOI:** 10.1038/s41426-018-0096-z

**Published:** 2018-05-25

**Authors:** Mohanraj Manangeeswaran, Jennifer L. Kielczewski, H. Nida Sen, Biying C. Xu, Derek. D.C. Ireland, Ian L. McWilliams, Chi-Chao Chan, Rachel R. Caspi, Daniela Verthelyi

**Affiliations:** 1 Division of Biotechnology Review and Research-III, Office of Biotechnology Products, Center for Drug Evaluation and Research, Food and Drug Administration, Silver Spring, MD 20993 USA; 2 Laboratory of Immunology, NEI, NIH Bethesda, MD 20892 USA

## Abstract

Zika-infected patients can have eye involvement ranging from mild conjunctivitis to severe chorioretinal lesions, however the possible long-term sequelae of infection and timeline to recovery remain unknown. Here we describe the partial recovery of chorioretinal lesions in an immunocompetent patient diagnosed with bilateral posterior uveitis associated with Zika infection and show that some lesions resolved with focal atrophy evident as pigmentary changes on funduscopy. To better understand the progression of the lesions and correlate the changes in fundus imaging with local viral load, immune responses, and retinal damage, we developed a symptomatic mouse model of ocular Zika virus infection. Imaging of the fundus revealed multiple hypopigmentary patches indicative of chorioretinal degeneration as well as thinning of the retina that mirror the lesions in patients. Microscopically, the virus primarily infected the optic nerve, retinal ganglion cells, and inner nuclear layer cells, showing thinning of the outer plexiform layer. During acute infection, the eyes showed retinal layer disorganization, retinitis, vitritis, and focal choroiditis, with mild cellular infiltration and increased expression of tumor necrosis factor, interferon-γ, granzyme B, and perforin. Focal areas of gliosis and retinal degeneration persisted 60 dpi. The model recapitulates features of ZIKA infections in patients and should help elucidate the mechanisms underlying the damage to the eyes and aid in the development of effective therapeutics.

## Introduction

Zika virus (ZIKV) belongs to the flavivirus family along with West Nile, Yellow fever, Dengue, and Japanese encephalitis viruses. Over the last 2 years, it has raised alarm as it infected people in > 70 countries causing serious malformations in newborn and neurological diseases in adults. Congenital ZIKV infection is associated with neurological, ocular, auditory, and skeletal abnormalities. In the eye, microphthalmia, chorioretinal lesions such as macular pigment mottling and atrophy in the area surrounding the macula, retinal vasculitis, hypoplasia of the optic nerve, and iridocyclitis have been reported in newborns of women infected during pregnancy^1–4^. Retinal abnormalities in the absence of microcephaly have also been reported^5^. In the eyes of adults, ZIKV presents most frequently as a non-purulent conjunctivitis; however, more serious findings including disruption of the outer macular retinal pigment epithelium (RPE), and iridocyclitis have been described in healthy and immunocompromised subjects^6–8^. At this time, the long-term consequences of these infections are unknown, but a report in an immunocompromised patient suggests that lesions can be persistent^7^. In addition, infectious virus has been isolated from conjunctival swabs of ZIKV-infected patients, demonstrating the ability of ZIKV to infect peri-ocular tissues and raising the possibility of transmission through ocular secretions^9^.

Recent in vitro studies show that ZIKV can infect the cells lining the inner blood–retinal barrier, the retinal endothelium, and RPE leading to cell death^10–12^; however, it was unclear whether systemic infections would result in ZIKV penetrating the BRB to infect the eye in vivo. Animal models that recapitulate the disease and recovery in humans, and replicate the damage caused by ZIKV infection in the eye can help elucidate the pathogenicity of the virus and the role of immune response in viral clearance and recovery. Adult mice are usually resistant to neurotropic virus possibly owing to the protection of the BBB and BRB, reduced susceptibility of mature neurons to infection, and an inability of ZIKV to block a robust interferon response, which can clear the virus^13^. Therefore, interferon-deficient young mice were initially used to model ZIKV disease. Using this model, Miner et al. showed that Zika-infected mice can develop conjunctivitis, panuveitis with virus present in the cornea, iris, optic nerve, and the ganglion and bipolar cells in the retina^14^. However, global impairment of interferon can modify the susceptibility of specific tissues to ZIKV infection and alter the host’s inflammatory response to the virus potentially altering the pathophysiology of the disease. Indeed, Zika-infected interferon-deficient animals succumb to disease early after infection^15–17^. To explore the host–pathogen interactions in an immune-replete in vivo system we recently developed a mouse model wherein neonatal C57BL/6 wild-type mice (B6wt) are infected 1 day post birth (p1) with the recent ZIKV isolate PRVABC59, isolated by CDC from an infected patient serum who traveled to Puerto Rico in 2015^18,20^. When infected subcutaneously (SC), these mice develop transient encephalitis 12–15 days post infection (dpi) that manifests as severe ataxia, unsteady gait, loss of balance, kinetic tremors, and seizures that subside 1–2 weeks later.

In the present study, we describe the presence of persistent atrophic chorioretinal lesions in a convalescent immunocompetent adult patient that developed bilateral posterior uveitis associated with Zika virus infection. We then use the B6wt model described above to show that subcutaneous infection can lead to a symptomatic posterior uveitis that recapitulates the infection in the patient. Using this model, we show that ZIKV preferentially infects the cornea and retina resulting in chorioretinal lesions. As reported in patients, there is infection of the retinal ganglion cells and cells in the inner nuclear layer, resulting in focal retinitis and thinning of the outer plexiform layer (OPL)^19^. The infection elicits an inflammatory response characterized by increased local chemokine expression, infiltration by neutrophils, antigen presenting cells, natural killer (NK) cells, and later CD4 and CD8 T cells. Interestingly, the lesions persist for several weeks after the neurological symptoms abate and viral loads clear as tissue damage is still evident 60 and 90 dpi. These studies show ZIKV infection of the immune privileged posterior eye can lead to long-lasting chorioretinal lesions.

## Results

### Persistent retinal lesions in a healthy ZIKV-infected adult with posterior uveitis

**Fig. 1:**
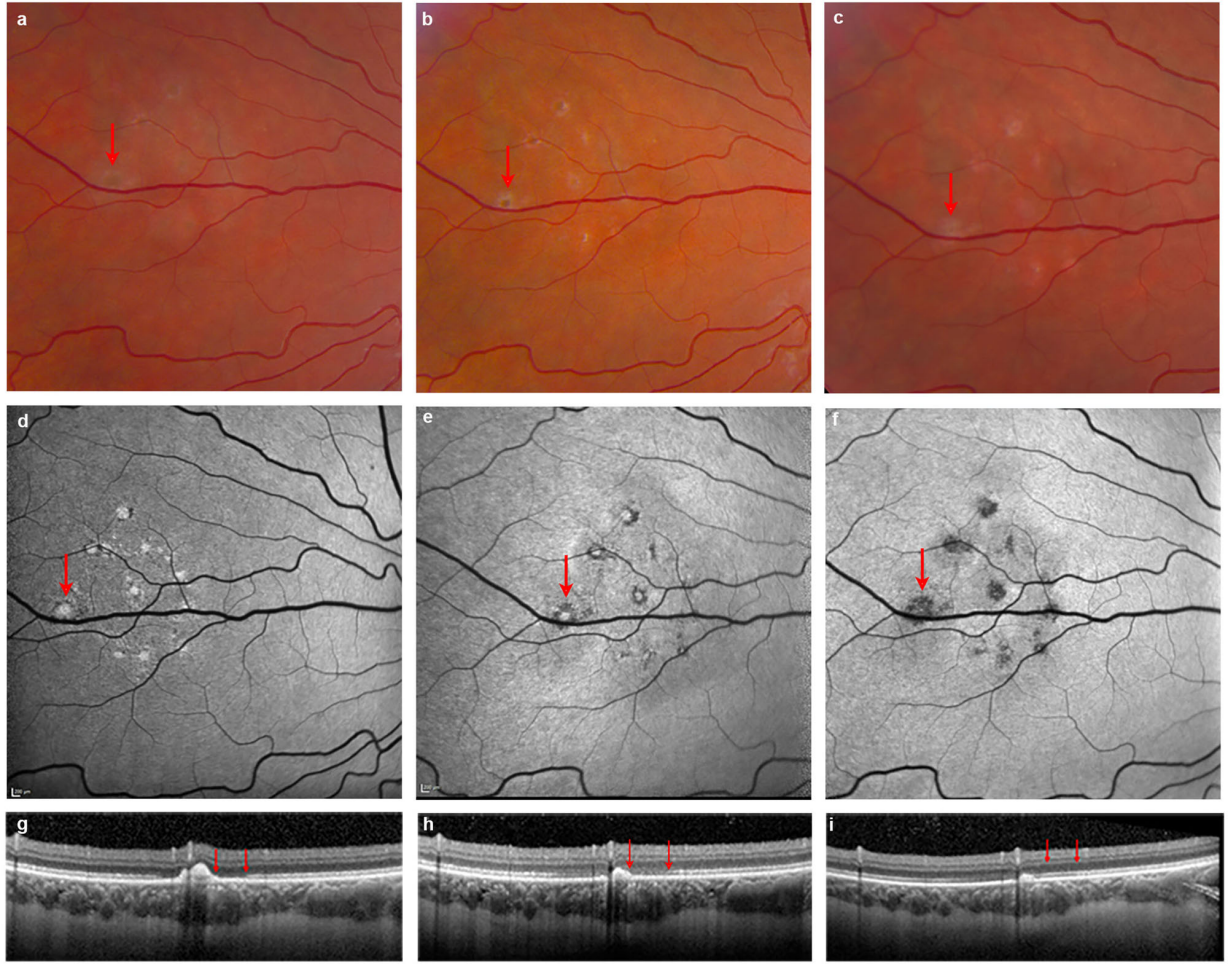
Posterior lesions in ZIKV-infected patient. **a**, **b**, **c** Fundus photos illustrate the evolution of outer retinal lesions with increasing pigmentation over time. **d**, **e**, **f** Fundus autofluorescence (FAF) images corresponding to outer retinal lesions show hyperautofluorescence at the initial presentation that becomes hypoautofluorescent by 5 months, indicating partial resolution with RPE atrophy. **g**, **h**, **i** Spectral domain OCT confirms the outer retinal location of lesions with nodular hyperreflective elevated spots corresponding to each hyperautofluorescent lesion. The OCT illustrated here represents images across the largest lesion (indicated by arrow on FAF, Fig. 1d). One and 5 months after onset there is spontaneous partial reconstitution of outer retina with resolution of some lesions, whereas some lesions result in atrophy. Note that the outer retina immediately adjacent to the largest lesion shows reconstitution of the ellipsoid zone by 5 months concurrently with the resolution of the nodular elevation on OCT (two red arrows). Note that images corresponding to onset and 1 month follow-up correspond to a previously reported patient^6^ and are provided here as reference for month 5

Although the acute effects of ZIKV have been described, the long-term consequences of infection are yet unknown. We recently reported a case of a healthy adult patient treated at the NIH who had developed symptoms of ZIKV after a trip to Puerto Rico^6^. He had developed floaters in his left eye ~ 2 weeks later without loss of visual acuity. This patient had a confirmed diagnosis of ZIKV by reverse transcriptase (RT)-PCR and was negative for Dengue and chikungunya viruses. Examination showed +0.5 grade (mild) cells in the vitreous and scattered mid peripheral yellowish–white deep retinal lesions in the nasal retina. The details of this case at presentation and 1 month follow-up have been previously reported elsewhere^6^ but, in brief, multimodal imaging showed a cluster of hyperautofluorescent lesions on fundus autofluorescence (FAF) in the nasal retina, which corresponded to hyperreflective nodular elevations on spectral domain optical coherence tomography (OCT). Separately, there were focal areas of presumed choroiditis visualized as hypercyanescent lesions on indocyanine green angiography. We now report that follow-up at 5 months after onset shows reconstitution of the outer retinal layers as visualized on FAF and OCT, however some lesions resolved with atrophy evident as pigmentary changes on funduscopy and hypofluorescence on FAF (Fig. 1).

### ZIKV infects the eyes of B6wt mice

**Fig. 2:**
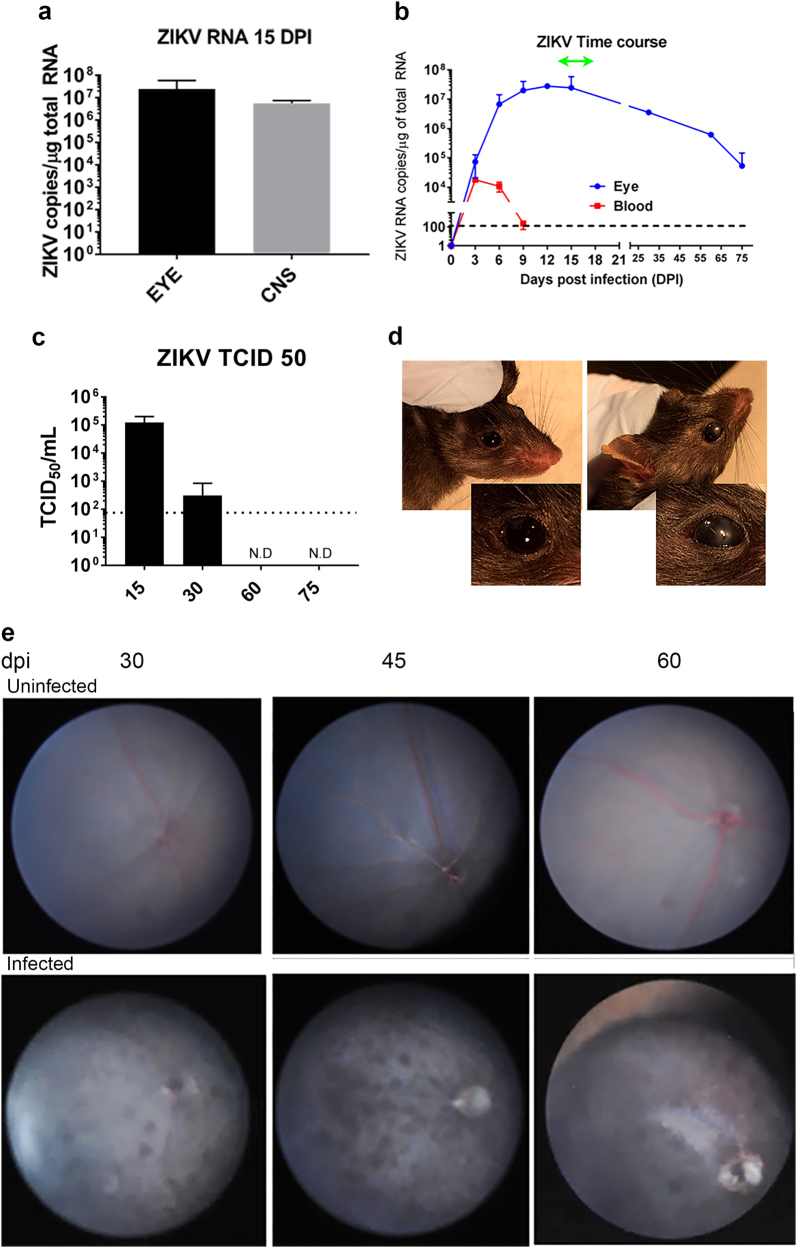
ZIKV infects the eye and causes chorioretinal lesions. **a** Levels of viral RNA in CNS and eye at 15 dpi (mean ± SD, *n* = 6; *p* = ns); **b** Levels of viral RNA in whole blood and eye homogenates over time. ZIKV RNA was measured in the blood, brain, and eye of B6wt mice (mean ± SD, *n* ≥ 3 mice/time point) using quantitative real-time PCR. Values are presented as number of viral RNA copies/microgram of total RNA. Green line represents the period when mice display ataxia and seizures^29^. **c** Levels of infectious ZIKV was measured by TCID_50_ assay using eye homogenates from ZIKV-infected mice (mean ± SD, *n* ≥ 3 mice/time point) at 30, 60, and 75 dpi. Dotted line represents lower limit of quantification of the assay. **d** Opacity of the cornea in ZIKV-infected (30 dpi, right) and age-matched control (left); overall incidence was 12%. Inset shows magnification of the eye; **e** Fundus image of uninfected age-matched controls (top panels) and ZIKV-infected (bottom panels) B6wt mice showing hypoautofluorescent lesions at 30, 45, and 60 dpi (representative of six mice/time point)

The development of an animal model that replicates the posterior uveitis described in ZIKV-infected patients would facilitate understanding the pathogenesis of the lesions. We previously showed that B6wt pups challenged SC with ZIKV develop a non-lethal encephalitis with onset between days 12 and 15 and resolution 7–10 days later^20^. To determine whether the ZIKV infection reaches the eyes in this model we sacrificed the mice at the peak of neurological disease (day 15), collected the eyes, and quantified ZIKV RNA. As shown in Fig. 2a, p1 mice challenged with ZIKV have similar levels of virus in the eyes as in the CNS on day 15, as assessed by viral RNA copy number. These data indicates that systemic infections with ZIKV in neonatal mice lead to ocular infections as previously reported for other TORCH (Toxoplasma gondii, Other, Rubella virus, Cytomegalovirus, and Herpes simplex virus) pathogens^20^.

To gain a better understanding of the dynamics of viral spread in our model, we next examined whether the peak concentrations of viral RNA in the eye corresponded to that of peripheral blood. Eyes were collected on 3, 6, 9, 12, 15, 30, 60, and 75 dpi. As shown in Fig. 2b, there were detectable levels of ZIKV virus RNA as early as 3 dpi, which is the earliest time point that viral RNA can be detected in the CNS (manuscript in preparation). This suggests that the eye infection may spread hematogenously to brain and the eye simultaneously, although it is not possible to discount transfer via the optic nerve. Moreover, day 3 coincides with the peak of ZIKV RNA in blood. Together, these findings support the hematogenous spread of the virus directly to the eye as previously suggested^21^. After day 3, the progressive increase in viral RNA levels in the eye is markedly different from that of peripheral blood (Fig. 2b), which suggests that although ZIKV is rapidly cleared from the periphery, it tends to persist in the eye tissues as reported for other pathogenic viruses^22^, possibly owing to ocular immune privilege. Given that the ZIKV-infected B6wt mice survive the infection, we next determined whether the eyes clear the infection after the symptoms of disease subside. Interestingly, although there is a progressive reduction in viral RNA after day 15, significant levels persisted in the eye at day 30, 60, and 75, which is 7–8 weeks after the clinical signs of the encephalitis have subsided (Fig. 2b). The presence of live virus was confirmed by TCID_50_ in eye homogenates at 15 and 30 dpi (Fig. 2c).

### ZIKV-infected mice develop chorioretinal lesion in the eyes

Having shown that neonatal B6wt mice challenged SC with ZIKV develop high levels of viral RNA in the eye, we next examined whether the infection was associated with lesions that resembled those in humans. Macroscopically, most eyes appeared normal, however 12% of infected mice (4 of 30) had asymmetric opacity of the cornea that were not evident in mock infected animals (Fig. 2d), although the role of ZIKV in this phenotype is unknown.

Fundoscopy is used to diagnose macular and perimacular pigmented atrophic lesions, diffuse RPE damage, and chorioretinal atrophy in the babies with post-natal ZIKV syndrome^23^. Similarly, there are reports of healthy and immunocompromised adults where the examination of the fundus revealed pigmented outer retinal lesions as well as hyperreflective elevations of the outer retina and loss of ellipsoid layer^6,24^. Because mice open their eyes around day 15, day 30 is the earliest time when a live fundoscopy could be performed, thus the fundi of infected mice were imaged at 30, 45, and 60 dpi (Fig. 2e). Interestingly, even though the neurological disease in ZIKV-infected mice waned by day 21 and the levels of viral RNA had declined by day 30, imaging of the fundus in B6wt mice showed multiple hyperpigmentary patches suggestive of chorioretinal degeneration as well as thinning of the retina that became more severe by day 45 (Fig. 2e). The presence of hyperpigmented lesions in the infected mice recalled those reported in adult patients^7^. Of note, the additional blurring of the imaging of the vasculature in the fundus that recalled reports in congenital ZIKV syndrome^23^. Interestingly, by 60 dpi, the lesions were reduced, but had not completely disappeared. Together, these data indicated that systemic infection of newborn mice leads to symptomatic infection of the eye with local inflammation and chorioretinal lesions that resemble those found in humans affording the possibility of assessing the histological and immunological changes underlying the lesions.

### ZIKV infection leads to local inflammation and cellular infiltration

**Fig. 3:**
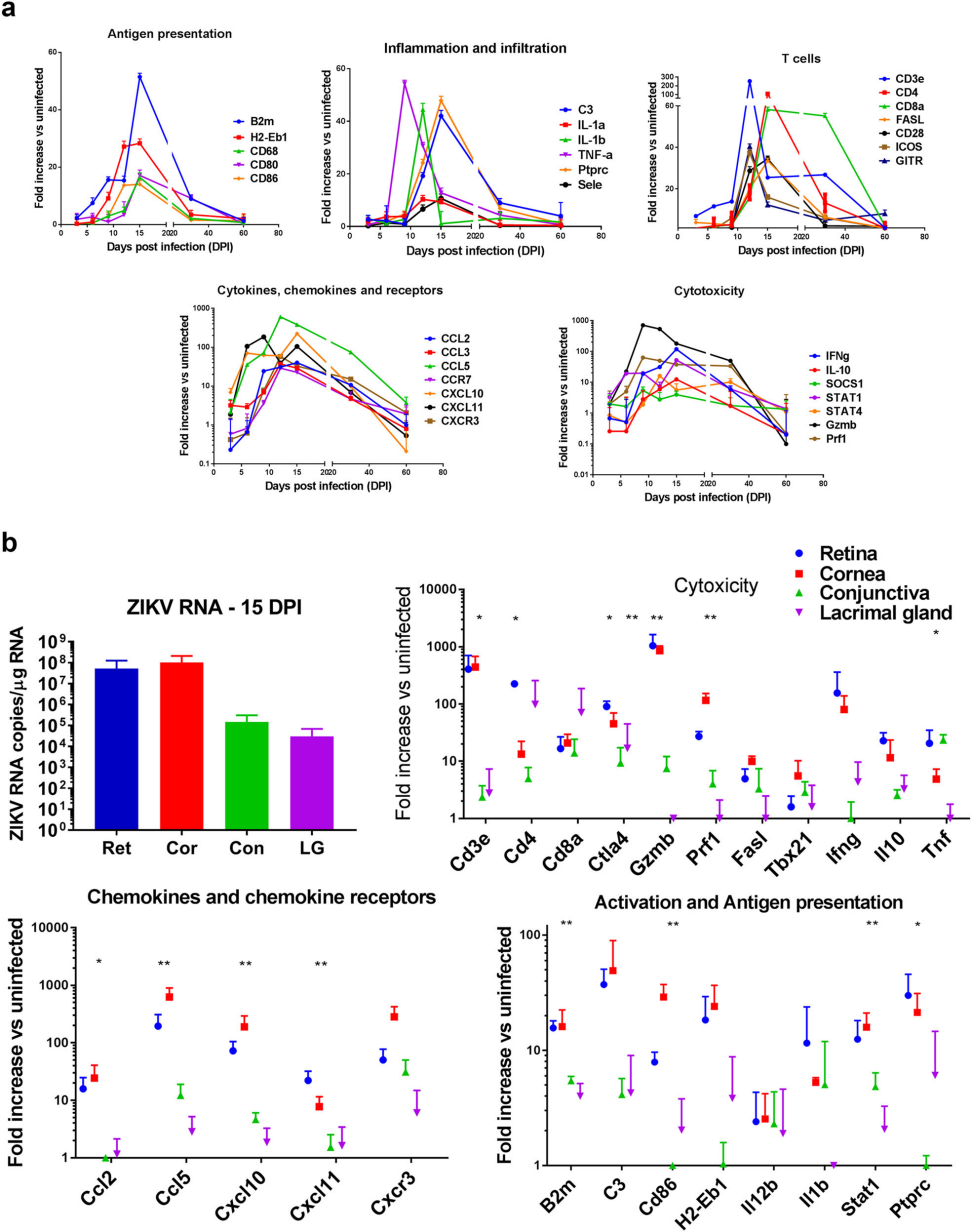
ZIKV induces inflammatory gene expression. **a** RNA was isolated from the eye homogenates of B6wt mice 3, 6, 9, 12, 15, 30, and 60 dpi. Relative expression of immune-related genes assessed using a TaqMan mouse immune array and shown as mean fold increase over uninfected, age-matched controls (*n* = 6/time point). **b** RNA isolated from retina, cornea, conjunctiva, and lacrimal gland 15 dpi and assessed for viral load by qRT-PCR as well as mRNA expression of immune-related genes. Gene expression was calculated as fold increase over uninfected, age-matched control samples of the same tissue. Mean ± SD, *n* = 3; Differences between tissues were tested by ANOVA *p < 0.05; **p < 0.01

The eyes are generally considered as an immune-privileged organ protected by the tight junctions of the blood–retinal barrier and mechanisms that actively downregulate immune responses in the eye to protect delicate ocular tissues from immune mediated damage. Thus, few immune cells reside within ocular tissue^25,26^. Upon infection however, peripheral immune cells can enter the eyes causing local inflammation^27^. To characterize the local response to the virus, we assessed the expression of 90 immune-related genes in the eyes at different stages of the disease. Although ZIKV RNA is detected in the eye by 3 dpi, the expression of immune-related genes lags. Indeed, at 3 dpi when infection peaks in plasma, there is only a mild increase in the gene expression of CCL3, CXCL10, and CXCL11 in response to the virus in ocular tissue and no evidence of immune cell infiltration. By 6 and 9 dpi, viral RNA levels increase to > 10^6^ copies/μg of total RNA, and the eyes show increased levels of local expression of STAT1, and chemokines CXCL10, CXCL11, and CCL5 (Fig. 3a) that suggest the activation of local glial cells and initiation of cellular recruitment. By day 9, there is a subtle increase in gene expression pertaining to MHC, B2m, and STAT1, suggesting the activation of local glia-like cells in the eye. In addition, there is an increase in TNFα, granzyme, perforin, and IFNγ that is not accompanied by increased expression of T-cell markers CD3 or CD8, suggesting that other cells, possibly NK cells, may reach the infected eye early in the disease. A few days later (days 12–16), when the mice develop clinical signs of encephalitis, the expression of chemokines in the eye peaks and there is a clear increase in CD45, as well as increased expression of MHC, CD80, CD86, and CD68. This coincides with a surge in expression of mRNA for IL1a, IL1b, C3, and selectin, suggesting the infiltration of monocytes. At that time there is also an increase in the expression of CD3, CD8, CD4, CD28, ICOS, GITR, and FasL together with higher levels of perforin1, granzyme B, IFNg, and IL-10 transcripts as compared with the uninfected eye. This suggests the presence of infiltrating T cells with a cytotoxic or Type1 profile. No increase in CD19, IL17, or FoxP3 was evident at any time during the course of the infection, suggesting that there is no significant infiltration by B cells, TH17, or Treg cells into the eye during infection. The presence of infiltrating T cells, macrophages, and dendritic cells at 15 dpi was confirmed by flow cytometry analysis (Supplementary Figure S1). Interestingly, the expression of most of the genes linked to inflammation and antigen presentation return to baseline by 30 dpi, however several chemokines remain highly expressed at day 30 as do CD3, CD8, prf1, and granzyme b, suggesting that cytotoxic T cells remain in the eye after the viral loads start to decline.

The eye is a complex and tightly compartmentalized organ. To better understand the response of each compartment in the eye, we isolated the cornea, conjunctiva, retina, and lacrimal glands at 15 dpi and quantified the local viral RNA. As shown in Fig. 3b, viral RNA is present in both anterior and posterior regions of the eye, however the cornea and retina have more than 100-fold higher viral RNA titers compared with the conjunctiva or lacrimal gland (Fig. 3b). This suggests that the virus predominantly targets ocular structures that are rich in neuronal cells. The more heavily infected tissues, cornea and retina, had higher levels of mRNA for chemokines as well as genes associated with infiltrating CD45 + cells including biomarkers linked to antigen presenting cells (CD86, B2m, H2-EB1) and T-cell infiltration (CD3, CD4, GITR, CD40L, Fas-L), as well as c3, IFNg, TNFα, granzyme B, and perforin suggesting higher cytotoxic activity in the more heavily infected areas. Together, these data indicate that the virus initially causes local expression of chemokines and cellular infiltration of the retina and optic nerve.

### ZIKV infects the optic nerve head and chorioretinal tissue

**Fig. 4:**
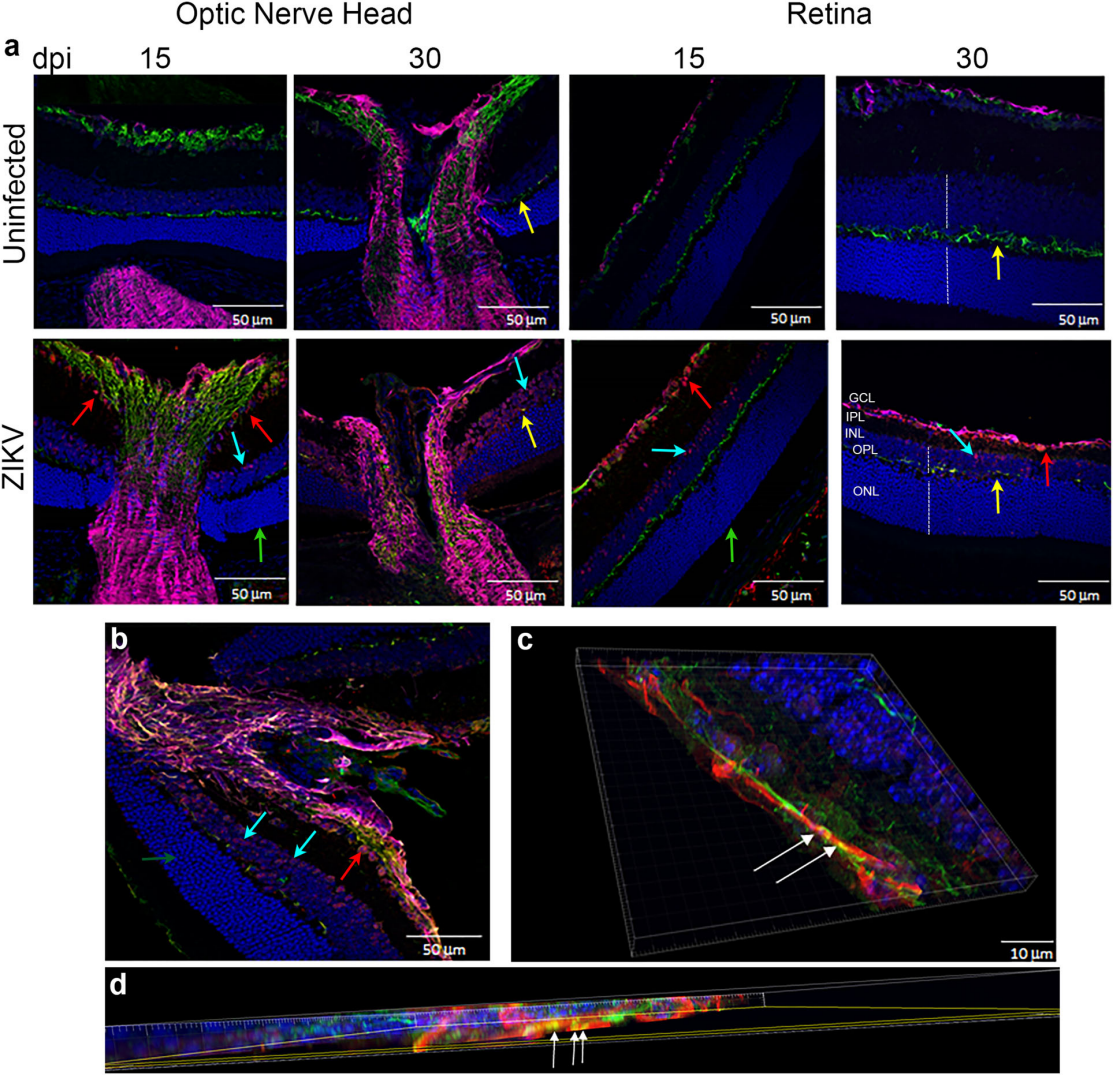
Optic nerve head and retina are infected with ZIKV. **a** IF-IHC staining of the optic nerve head and retina with antibodies to DAPI (blue), NF160 (green), GFAP (magenta), and EVU-302 (anti-ZIKV, red) at 15 and 30 dpi. **b** Higher magnification of optic nerve head at 30 dpi. **c**, **d** Confocal imaging of optic nerve head showing colocalization of virus and neuronal staining (white arrows). Red arrow shows virus infection of ganglion cells, Blue arrows show infected cells in inner nuclear layer (INL), green arrow shows uninfected photoreceptor layer (cones and rods), yellow arrows show the outer plexiform layer. Note this layer is reduced in the retina of infected mice. Dotted white lines show the width of the INL and ONL. Note the relative reduction in INL in infected mice at 30 dpi. Images representative of two independent experiments with three mice/group. Note that ZIKV antigen was in 100% infected mice at 15 dpi and 80% of infected mice at 30 dpi. Only 20% of infected mice were positive for ZIKV antigen at 60 dpi. ONL- Outer Nuclear Layer; OPL-Outer Plexiform Layer; INL-Inner Nuclear Layer; IPL-Inner Plexiform Layer; GCL-Ganglion Cell Layer

Correlating the changes in fundoscopy with the progression of the local immune response and architectural changes in the tissue can improve our understanding of the disease. To define the localization of the virus within the eye and determine whether the infection leads to changes to ocular morphology, we stained eye sections with antibodies specific to the virus (antibody clone EVU-302), neurons (anti-neurofilament 160), and glial cells (GFAP) at 15, 30, and 60 dpi. As shown in Supplementary Figure S2, ZIKV is evident in the optic nerve head and ganglion cells of the retina by 15 dpi. The optic nerve head harbors the highest concentration of viral antigen (stained in red Fig. 4 and Supplementary Figure S2) and there is significant colocalization with neuronal fibers (white arrows, Fig. 4c, d), although astrocytes also stained positive for ZIKV infection (Fig. 4a, b and not shown). In the retina, the virus stained most notably the inner nuclear layer (INL) likely the amacrine and bipolar cells (blue arrows) and retinal ganglion cells (red arrows, Fig. 4a, b). Interestingly, no staining was evident in the cones and rods of infected mice (Fig. 4a, b green arrow). Viral antigen was still evident at day 30, but declined by 60 dpi (Supplementary Figure S2). Interestingly, relative to 15 dpi, at 30 dpi the eyes display more significant morphological changes as the optic nerve shows signs of increasing gliosis and foci of atrophy (Supplementary Figure S3), evident as thickening of GFAP + processes, and the retina displays a loss of cells in the INL and retinal ganglion cells resulting in reduced thickness of the OPL (Fig. 4a, yellow arrows). Similar to 15 dpi, virus staining of rod and cone photoreceptor cells was negative at day 30 confirming that these cells are not infected in this model. The virus infects both the anterior and posterior areas of the eye leading to a narrowing of the retinal INL and apparent loss of ganglion cells with reduced thickness of the outer plexiform layer^6,7^. At last, at day 60, although viral RNA could still be amplified from the tissue, live virus could not be found by TCID50 and IHC shows markedly reduced levels of viral antigen in the optic nerve and retina as compared with 15 or 30 dpi, suggesting that the virus is being cleared from the tissues (Fig. 2c and Supplementary Figure S2).

### ZIKV infection causes mild vitritis and focal chorioretinitis

**Fig. 5:**
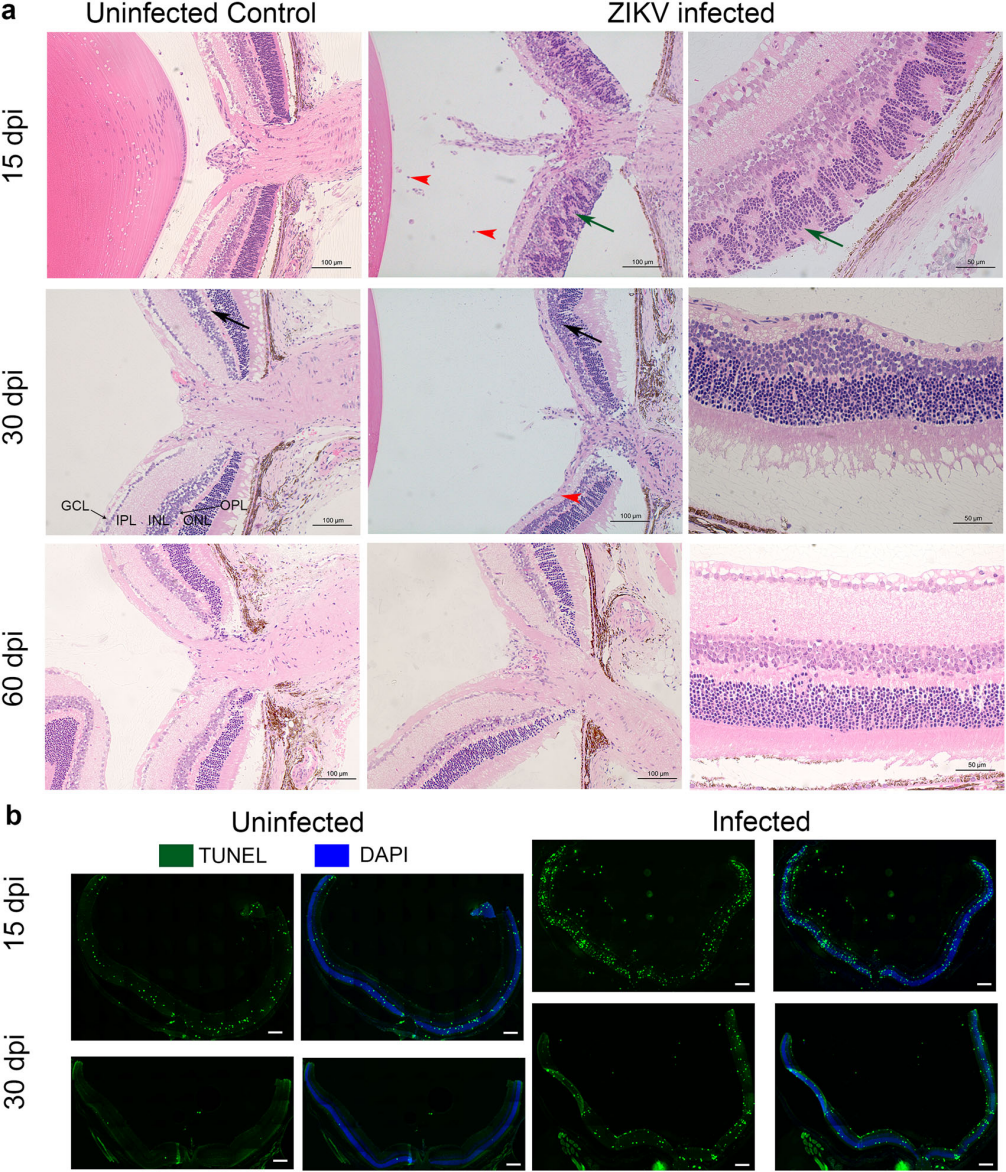
ZIKV infection induces apoptosis and damages the architecture of the eye. **a** Histopathology of ZIKV infected Eyes shows distinct retinal folding, vitiritis and choroiditis. H&E staining of sections from B6wt eyes harvested 15, 30 and 60 dpi. Left panels show uninfected age-matched controls. The center panels show histopathology at 15, 30, and 60 days post infection. Right panels show a magnified image of the infected eye of the same mouse. Black arrows indicate the reduction of thickness of the outer plexiform layer. Note that the width is restored at 60 dpi. Green arrows indicate the retinal folding. Red arrowheads point to cellular infiltration. ONL- Outer Nuclear Layer; OPL-Outer Plexiform Layer; INL-Inner Nuclear Layer; IPL-Inner Plexiform Layer; GCL-Ganglion Cell Layer. **b** Apoptotic cells stained with TUNEL (green) and DAPI (Blue) at 15 and 30 dpi. Scale bar: 200μm. A higher magnification of the apaptosis can be found in Supplementary Figure S5. Images representative of two independent experiments with three mice/group

To better characterize the morphological changes in the eye that were evident by fundoscopy and IHC, we assessed the effect of the infection on the cellular architecture of eyes at 15, 30, and 60 dpi using H&E staining. As shown in Fig. 5a, at 15 dpi there is extensive disorganization of the retinal layers with distinct retinal folding and modest infiltration of inflammatory cells in the retina and vitreous. Infiltrating cells were also evident at the optic nerve head and within the uveal layers showing evidence of vitritis, retinitis, choroiditis, and iridocyclitis in the infected eyes. At 30 dpi (Fig. 5a) there is thinning and disappearance of the OPL, suggesting localized loss of neuronal synapses in this layer. Staining for cells undergoing apoptosis confirms increased cell death in the infected retinas at 15 and 30 dpi, but not at 60 dpi (Fig. 5b and data not shown). Interestingly, despite significant reductions in viral RNA and expression of immune-related genes, lesions take weeks to heal and there is evidence of residual gliosis.

**Fig. 6:**
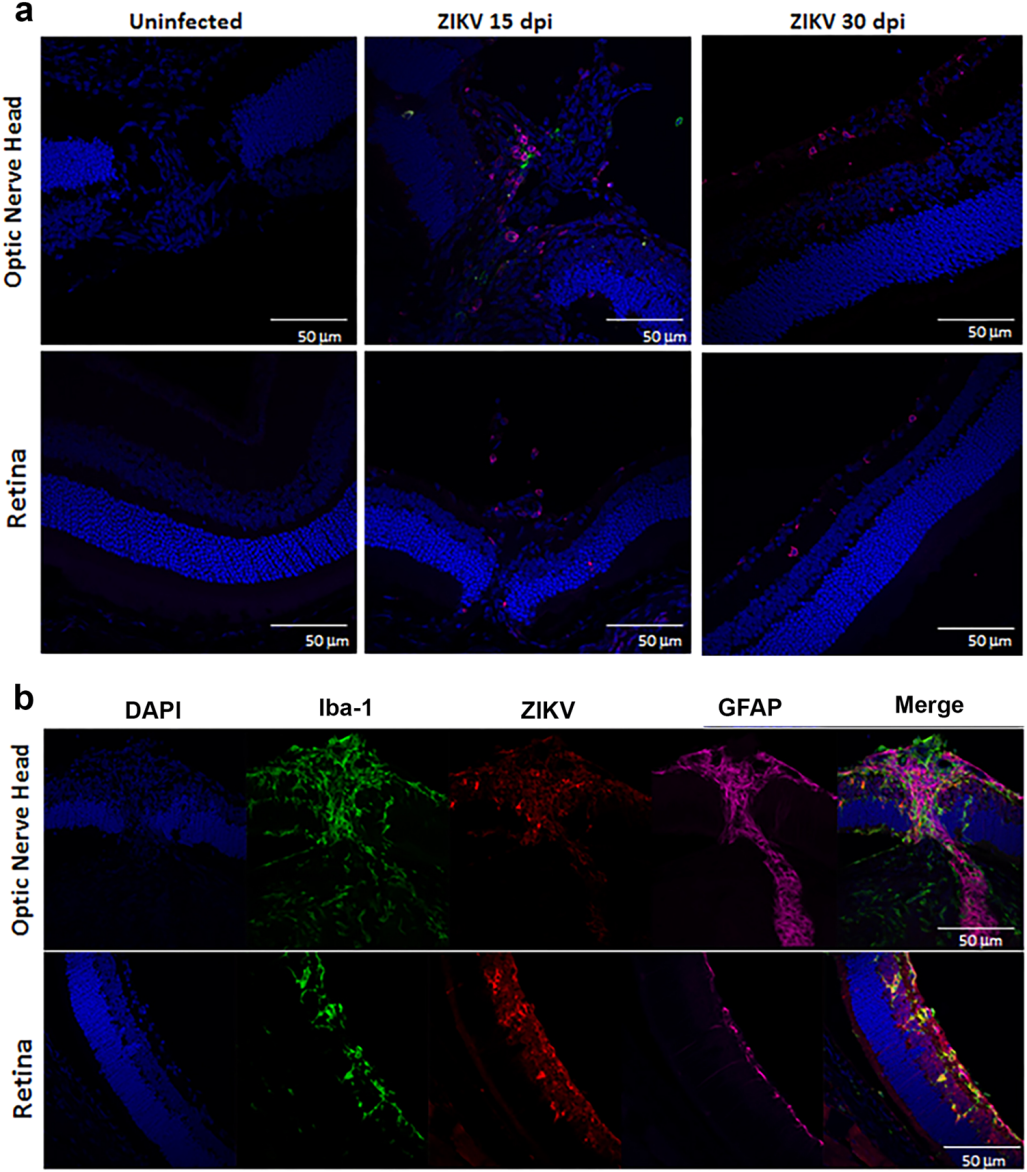
Inflammation and cellular infiltration in ZIKV infected eyes 15 and 30 dpi. **a** Infiltration of CD4 and CD11c + cells 15 and 30 dpi. Staining with DAPI (blue), anti-CD4 (red), anti-CD11c (green). **b** ZIKV colocalizes with microglia 15 dpi. Staining with DAPI (blue), Iba-1 (green), EVU-302 (red), GFAP (magenta) shows virus and Iba^+^ microglia are evident in the optic nerve and retina. Note colocalization of Iba-1 and EVU-302 and gliosis of surrounding area. Figure representative of three mice/time point with similar images

To confirm the presence and distribution of infiltrating cells, we stained the infected eyes (15 dpi) with anti-CD4 antibodies. As shown in Fig. 6a, the infected eye had infiltrating T cells in the optic nerve head and retina at 15 dpi. The T-cell infiltration was still significant at 30 dpi (but diminished by day 60, data not shown). This is accompanied by an increase in Iba-1 + cells that colocalize with the virus indicating activation of local glial cells and possibly cellular infiltration by APC (Fig. 6b and Supplementary Figure S4). These data are consistent with the increase in T cells, macrophages, and dendritic cells observed by Flow cytometry (supplementary Figure S1).

### Resolution of posterior uveitis in B6wt mice

**Fig. 7:**
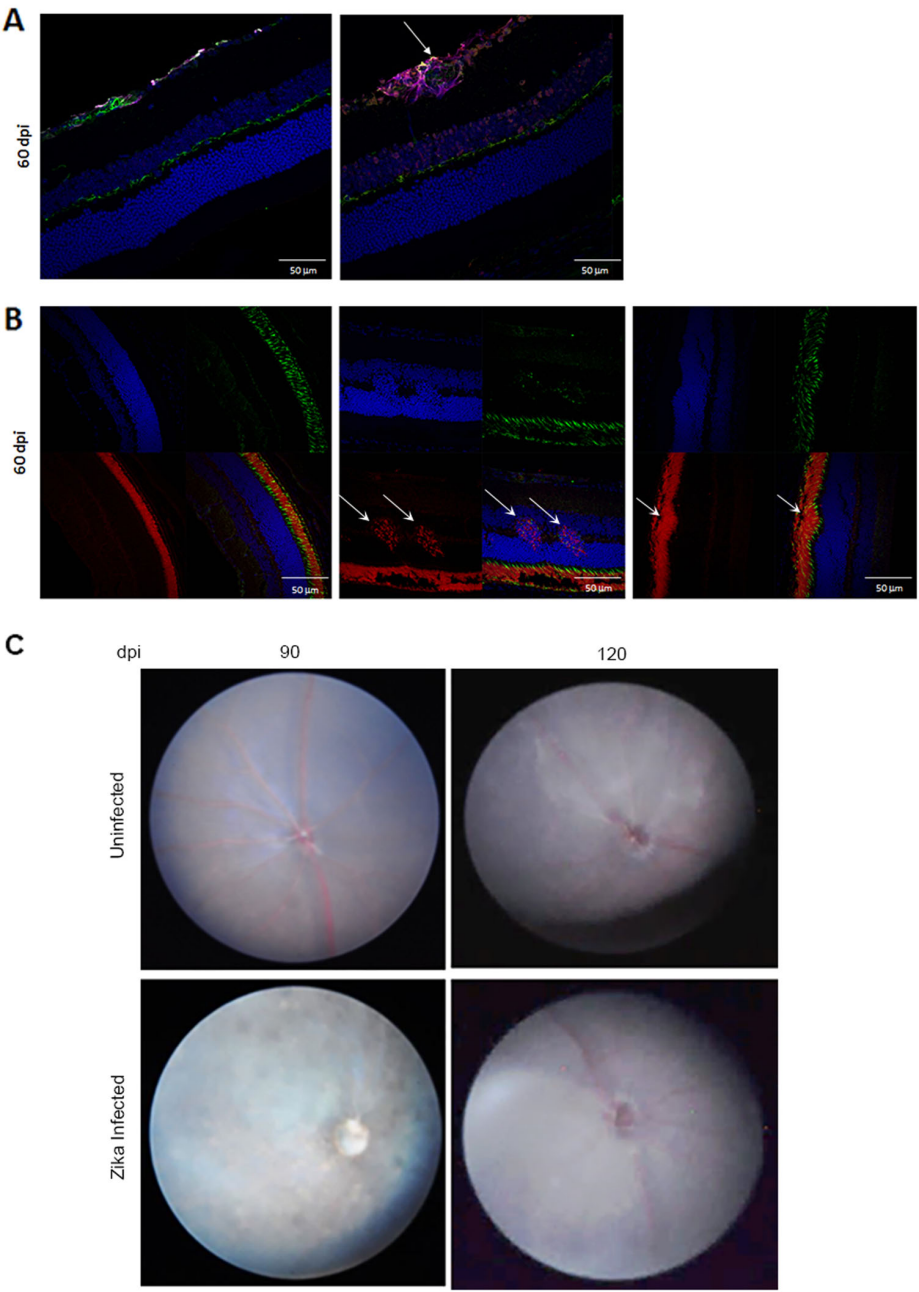
Lesion persistence in ZIKV infected mice. **a** IHC of retina at 60 dpi stained with DAPI (blue), NF160 (green), GFAP (magenta), and EVU-302. Image shows foci of gliosis that is not stained with EVU-302 indicating absence of viral antigen. **b** IHC of retina at 60 dpi stained with DAPI (blue), Wheat Germ Agglutinin (WGA) to stain rods (red), and PeaNut Agglutinin (PNA) to stain cones (green). Note that evidence of retinal degeneration (white arrows) modifies the photoreceptor layer. **c** Fundus imaging of ZIKV infected (bottom panels) and corresponding uninfected age-matched controls (top panels) at 90 and 120 dpi. Note that the vasculature is evident at 120 dpi. *N* = 3 mice per group

As B6wt mice infected with ZIKV survive the infection, it is possible to use the model to explore the resolution and long-term consequences of the infection. We showed above that viral RNA as well as the cellular infiltration started to decline after 30 days, and were markedly reduced by 60–75 dpi. Indeed, in most mice, histological examination of the eyes shows only minimal residual retinal folds and rare inflammatory cells at the optic nerve head at 60 dpi. These findings were at odds with the evidence of hypopigmentary lesions in the fundoscopy (Fig. 2), however, careful examination of the retina at 60 dpi identified isolated foci of gliosis and retinal degeneration. Together these data suggest that ZIKV infection results in persistent chorioretinal lesions. To determine whether lesions resolved over time we imaged the posterior eye of mice 90 and 120 dpi. As shown in Fig. 7, at 90 dpi there were still signs of disease with some minor hypopigmented lesions and blurred imaging of the vasculature, but these were no longer evident at 120 dpi. Overall, these results suggest that in our model ZIKV induces persistent inflammation and chorioretinal lesions that may heal months after the infection.

## Discussion

Viral infections may linger in immunoprivileged sites like the central nervous system or the eye. The recent description of a patient recovering from Ebola, where the blood and urine tests were negative but ocular inflammation including anterior uveitis and vitritis persisted, calls attention to the long-term effects of ocular infections and the role they might play in the infectious cycle^28^. Therefore, understanding the dynamics of ZIKV spread and clearance in the eye is important as it can inform as to (1) the possibility of viral persistence in potential reservoirs; (2) the optimal modality of treatment and the time to intervene to minimize lesions; and (3) whether the infection can lead to long-term sequelae predisposing patients to retinopathies later in life.

Recent reports suggest that patients infected with ZIKV can develop posterior uveitis^6,7,24^. Here we describe the evolution of ocular lesions in a healthy 26-year-old patient that had developed pigmented outer retinal and choroidal lesions and cellular infiltrates following ZIKV infection. Early in the disease the patient had shown a hyperreflective nodular elevation of the outer retina with an encircling loss of the ellipsoid layer. Although the patient regained visual acuity soon after infection, a follow-up visit 5 months after onset showed partial reconstitution of the outer retinal layers with evidence of persistent atrophic lesions. This suggests that the lesions in the posterior eye can persist after visual acuity has been recovered and that infection may result in chronic scarring of the retina. These results are consistent with those of Henry et al.^7^ who recently described a immunocompromised 60-year-old woman infected with ZIKV who developed bilateral diffuse, subretinal, confluent, placoid, and multifocal chorioretinal lesions. This patient’s visual acuity recovered within 6 weeks but retinal lesions required 5 month to heal.

Owing to unprecedented efforts to contain the disease, there are several candidate therapeutics and vaccines in various stages of clinical trials, but currently no FDA approved treatment or vaccine for ZIKV available. It is known that mice deficient in interferon response have altered susceptibility and various cells types of the eye can be infected. Development of non-immunosuppressed animal models that recapitulate the disease in humans is key to (1) explore host–pathogen interactions, (2) understand the role of the immune system in clearing the virus and in the pathogenesis of the lesions, and (3) select and develop therapeutic and vaccine candidates. It is well established in the literature that ZIKV does not replicate efficiently in adult immune competent mice. Initial studies using IFNAR^−/−^ mice or B6wt mice treated with anti-IFNAR antibodies had shown that ZIKV RNA can persist in several tissues, including the eyes, brain, and spleen long after the virus was cleared from the serum, however the immune response and thus susceptibility to the virus is altered in these mice^14,20,30^. Neonatal mice are naturally susceptible to ZIKV infection^29,31^. An immature immune system and an abundance of neuronal progenitors may contribute to the increased susceptibility to ZIKV, although it should be noted that Balb/c mice are not susceptible to disease, even when infected at day 1, suggesting that there are multiple factors contributing to susceptibility.

Previous studies have shown that lethal ZIKV challenges of neonatal wild type or IFNARKO mice can lead to ocular infections and cells of the inner nuclear layer, ganglion cells, and RPE cells can be infected^14,21^. Of note, assessment of the fundus in IFNARKO-infected mice did not show chorioretinal lesions^14^, suggesting that interferons and/or immune response may have a role in the development of ZIKV induced chorioretinal lesions. Chorioretinal lesions had only been observed in adult animals following high-dose intravitreal infections^11^. In this study, we have built on those findings to demonstrate for the first time that a systemic non-lethal ZIKV infection can lead to chorioretinal lesions similar to those in humans and followed their progression to document the persistence of lesions long after the neurological disease subsided.

In these mice, a SC infection results in detectable levels of viral RNA by 3 dpi both in the eye and in the brain. The spread of the virus into the eye could be either hematogenic or axonal but the surge of virus at the peak of viremia suggests that the initial seeding of these organs is hematogenic possibly via the highly vascularized uvea. The optic nerve becomes heavily infected and virus can be found in the occipital lobe later in the disease, suggesting that axogenic spread could take place as well (manuscript in preparation). Imaging of the eye shows that the virus infects not only the optic nerve, but spreads throughout the retina infecting the ganglion, bipolar, amacrine, and Muller cells in the inner nuclear layer, but not the photoreceptors. This is consistent with previous observations by Singh et al.^11^, who tested human primary and immortalized RPE, vascular endothelial, retinal Mϋller glia, and choroidal endothelial as well as photoreceptor cell lines and showed that photoreceptor cells were less permissive to the virus^33^.

Even though the photoreceptor cells are not infected, during disease there is increase in retinal folding and edema that distorts the architecture of the retina, particularly the inner nuclear layers^32^. The molecular and cellular basis for the folding is still unclear but may involve changes to the attachment of the RPE and the retina^33^. These pseudorosettes formed in the outer retina have been previously reported in retinal degeneration resulting from perinatal insult by other virus or chemicals^33^. In addition, histological and funduscopic examination of the eyes showed thinning of the OPL that resembled the neurosensory retinal thinning reported in adult patients infected with ZIKV^5,6^. The OPL is a layer of dendrites of the horizontal cells and bipolar cells of the inner nuclear layer that form synapses with the photoreceptor cells. It is possible that the increased apoptosis of infected cells in the inner nuclear layer may contribute to the thinning of the OPL. Future studies will need to address whether the virus induces a disruption of the vascularization of the retina in this model.

The eye is usually protected from the immune system by various factors, including a tight blood–retinal barrier, immunosuppressive cytokines such as TGF-β, inhibition of complement activation, and expression of FasL^25^. However, during ZIKV infections there is evidence of mild cellular infiltration, increased cytotoxicity, and inflammation in patients (mostly reported as 0.5 + cells)^6,7,24^. As shown above, infection of B6wt mice leads to a modest cellular infiltration, mainly by neutrophils and CD11c + dendritic cells in the vitreous humor, whereas T cells appear to predominate within the retina (Figs. 5 and 6). This mild infiltration is consistent with the increased expression of several chemokines early in the disease followed by increased levels of CD3, CD4, and CD8 as well as sustained increases in the levels of IFNγ, STAT1, Granzyme B, and perforin mRNA that suggest a cytotoxic process. This cellular infiltration is accompanied by gliosis and increased expression of Iba-1 and GFAP in the infected areas (Fig. 6b and supplementary Figure S3 and S4). The inflammation and infiltrating cells may play a key role in clearing the virus but may also contribute to the development of lesions. Future studies will assess the role of the adaptive immune system in lesion development and clearance as this may impact on possible therapeutic approaches.

Our studies confirm the persistence of virus in the eye despite an immunocompetent milieu beyond the clinical signs of disease and show that low levels of viral antigen and viral RNA can be found in the eye as late as 75 dpi, which is 6–8 weeks after the clinical signs of encephalitis have subsided^28^. Importantly, although infectious virus cannot be recovered from the eyes beyond 30 dpi, the virus RNA is cleared by week 53 (not shown), and the integrity of the retina including the thickness of the OPL is restored, fundoscopy shows evidence of retinal lesions as late as 90 dpi (Fig. 7). This suggests that antiviral treatment may not be effective late in the disease and the timing and treatment should aim to minimize sequelae. Understanding the role of the immune response in the generation and persistence of the retinal lesions may inform the utility of using immune suppressors such as corticoids late in the disease. In summary, this is the first study that shows peripheral ZIKV infection in a naturally susceptible host can lead to a productive infection of the eye with degeneration of the retina resulting in persistent chorioretinal lesions.

## Materials and methods

### Patient

A 26-year-old healthy white male developed constitutional symptoms (chills, myalgia), arthralgia, and skin rash 1 day after his return from his trip to Puerto Rico was seen under an IRB approved clinical protocol at the National Eye Institute (NEI). ZIKV infection was confirmed by RT-PCR assay from serum and RT-PCR assays were negative for Dengue and chikungunya viruses. Patient’s clinical records and longitudinally obtained ophthalmic images were reviewed.

### Mice

C57BL/6 (B6wt) mice were bred in AAALAC accredited, pathogen-free animal facility at the US food and drug administration. All experimental protocols were reviewed and approved by the Food and Drug Administration Animal Care and Use Committee (FDA-ACUC).

### ZIKV

Contemporary ZIKV isolate PRVABC59 generated by CDC in 2015 was used to infect the mice. This strain was isolated from the serum of a ZIKV-infected patient who travelled to Puerto Rico^18^. The complete genome sequence is published (Genebank accession #KU501215). Virus stocks from CDC were kindly provided by Maria Rios (FDA). This stock provided by CDC was used without any further passages in cell culture.

### ZIKV infections

All newborn mice were born from pathogen-free parents and inoculated with 2000 PFU ZIKV (50 μL) by intrascapular SC inoculation. B6wt mice were inoculated 1 day after birth (p1). For experiments tracking survival following ZIKV infection, mice were monitored daily for clinical signs of pathology for 30 days and twice weekly thereafter. All procedures were performed in accordance with the FDA-ACUC guidelines.

### RNA extraction, RT-PCR, and Taqman low-density arrays (TLDA)

Eyes were collected from infected animals that were perfused with 10 ml ice-cold phosphate-buffered saline (PBS). The eyes were flash frozen in liquid N_2_ and stored at −80 °C. The frozen tissue was homogenized in 1 mL of Trizol reagent (ThermoFisher, Carlsbad, CA) and RNA was isolated following the manufacturers’ protocol and resuspended in molecular grade ultra-pure ddH_2_O. To eliminate potential genomic DNA contamination, the DNA-free Turbo kit (ThermoFisher, Carlsbad, CA) was used as per the manufacturer’s protocol. The concentration and purity of isolated RNA was determined by spectrophotometry at 260 nm and 280 nm using a NanoDrop 1000 spectrophotometer (ThermoFisher, Carlsbad, CA). Reverse transcription was performed on 1 μg of total RNA, using Multiscript High Capacity Reverse Transcriptase (ThermoFisher, Carlsbad, CA), per the manufacturer’s protocol, using random primers. The resulting cDNA was diluted fivefold with ultra-pure water and stored at −20 °C prior to use in real-time Taqman PCR reactions (ThermoFisher, Carlsbad, CA).

Mouse Immune Array TLDA cards (TaqMan Mouse Immune array v2.1, ThermoFisher, Carlsbad, CA) were used as per manufacturers’ instructions. In brief, the cDNA generated above was diluted fourfold. Equal volumes of diluted cDNA and 2 × Universal Taqman Master Mix was prepared. This mixture was loaded into the chambers of a Taqman Array card, which was centrifuged to distribute the cDNA throughout the card. Real-time PCR acquisition and analysis was performed using a Viia7 real-time PCR machine with Quant Studio software, using automatic threshold and endpoint settings (ThermoFisher, Carlsbad, CA). Fold change in gene expression was determined using the ΔΔCt method^34^ with expression normalized using housekeeping gene GAPDH. Gene expression is shown as fold change relative to the indicated uninfected controls.

### ZIKV quantification

ZIKV RNA levels were measured using quantitative one step reverse transcriptase PCR to amplify ZIKV genome position 1087 to 1163 (GenBank accession no. AY632535). The assay can detect Asian and African genotypes of ZIKV but does not cross react with closely related flaviviruses and was performed as described^35^. The primer probe set used in this study has been demonstrated in previous studies to recognize PRVABC59 strain of ZIKV^29^. ZIKV RNA levels in the samples were quantified by comparing to a standard curve generated using dilutions of an in vitro RNA transcript copy of ZIKV sequence. We used 1 µg of total RNA for each sample analyzed and ZIKV RNA levels are expressed as ZIKV copies/µg of total RNA. In studies assessing different regions (tissues) of the eye, we used 1 µg of total RNA from the corresponding tissue.

For Tissue culture Infectious Dose 50 assay, eye homogenates were prepared from infected mice killed by CO_2_ asphyxiation and exsanguinated by trans-cardiac perfusion. Infectious ZIKV levels were measured as TCID_50_/0.5 g of tissue on Vero monolayers using an endpoint dilution assay as previously described^29^.

### Antibodies

The following antibodies were used for confocal microscopy. Primary antibodies to anti-CD4-alexa fluor 660 conjugated (eBioScience), CD11c-FITC conjugated (eBioscience), anti-ZIKV EVU-302 (Kerafast), anti-GFAP-alexa 633 conjugated (eBioscience), anti-NF-160 (Sigma-Aldrich), and anti-Iba-1 (Wako Chemicals).

### Immunohistochemistry

Eyes were enucleated from perfused ZIKV infected mice or age-matched uninfected control mice. The eyes were fixed in 4% paraformaldehyde for 2 h and washed in PBS prior to cryopreservation in 20% sucrose and Tissue-Tek O.C.T. (Sakura-Finetek, Torrance, CA). The tissue blocks were stored at −80 degrees until cut into 12 micron sections and placed on SuperFrost Plus slides (Fisher Scientific, Carlsbad, CA). The slides were stored at −80◦C degrees until use. Frozen retinal sections thawed at room temperature, washed in PBS for 10 minutes, and then blocked with 10% normal goat serum and 0.03% Triton-X 100 at room temperature for 1 h. The blocked tissue was washed with PBS for 10 minutes and stained with primary antibodies (1:25–1:200 dilution) overnight in blocking solution at 4 °C in humid chambers to prevent the slides from drying out. The sections were then washed with PBS three times for 10 minutes. If the primary antibody was not directly conjugated to a fluorochrome, then an AlexaFluor-568 or 488 secondary antibody (raised in goat) (Molecular Probes) was applied at 1:500 dilution along with DAPI (Invitrogen) at 1:500 dilution at room temperature. The slides were then washed three times in PBS and mounted with ProLong Gold (Molecular Probes). ApopTag Fluorescein in situ apoptosis detection kit (Catalog # S7110, Millipore, Massachusetts, USA) that uses TUNEL assay as the basis was used to detect cells undergoing apoptosis in ZIKV-infected eyes.

### Confocal microscopy and imaging

Retinas were imaged and processed using a Zeiss confocal laser-scanning microscope with Airy Scan (LSM 880) with Zen software (Carl Zeiss, Thornwood, NY). Confocal microscopy was performed with set parameters for laser power, photomultiplier gain, and offset, with a pinhole of diameter of 1 Airey unit for all samples with a × 40 oil immersion objective. Images of Z-stacks (10–15 optical sections with 0.5–1 μm step size) were acquired and maximum intensity projections of the Z-series were processed in Zen software. All images were compiled for publication in Adobe Photoshop CC 2015 software.

### Histopathology

Eyes were enucleated from exsanguinated ZIKV-infected mice or age-matched uninfected control mice as described above. Eyes were fixed in 10% formalin for at least 24 h. They were then embedded in methacrylate. The eyes were serially sectioned in the pupillary-optic nerve plane. All sections were stained with hematoxylin and eosin.

### Retinal imaging

Mice were anesthetized using ketamine/xylazine and were dosed based on body weight. Mice were kept warm by using heat pads or heat lamps. Immediately after injecting anesthetic, one drop each of phenylephrine hydrochloride ophthalmic solution (2.5%) and Tropicamide ophthalmic solution (0.5%) was added to each eye. A digital camera connected to an endoscope for taking pictures of mouse fundus was used. We placed a drop of hypromellose ophthalmic demulcent solution (2.5%) before flattening the eye slightly to view the fundus through the endoscope. After the exam, a drop of polyvinyl alcohol (1.4%) was placed on each eye to keep the eyes moist until the mouse awakens.

## Electronic supplementary material

Supplemenraty Figure S1

Supplementary Figure S2

Supplementary Figure S3

Supplemental Figure S 4

Supplementary Figure S5
