# Supplementary figures and images for: ZIKA virus infection causes persistent chorioretinal lesions
Source: Emerg Microbes Infect. 2018 May 25;7:96. doi: 10.1038/s41426-018-0096-z (PMC5970181; doi:10.1038/s41426-018-0096-z)

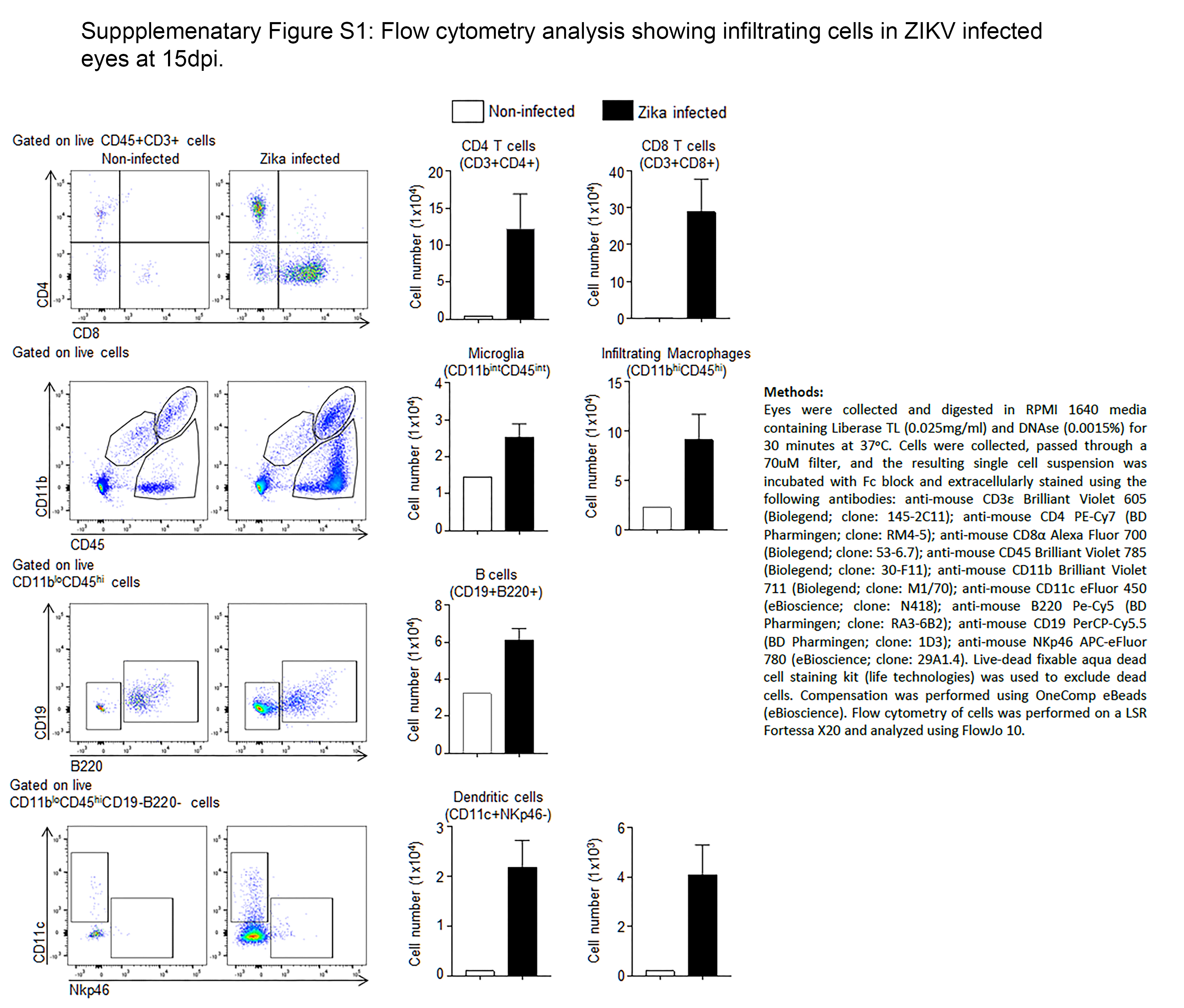

Supplement: Supplementary file 1 — Supplemenraty Figure S1 [file 41426_2018_96_MOESM1_ESM.jpg]

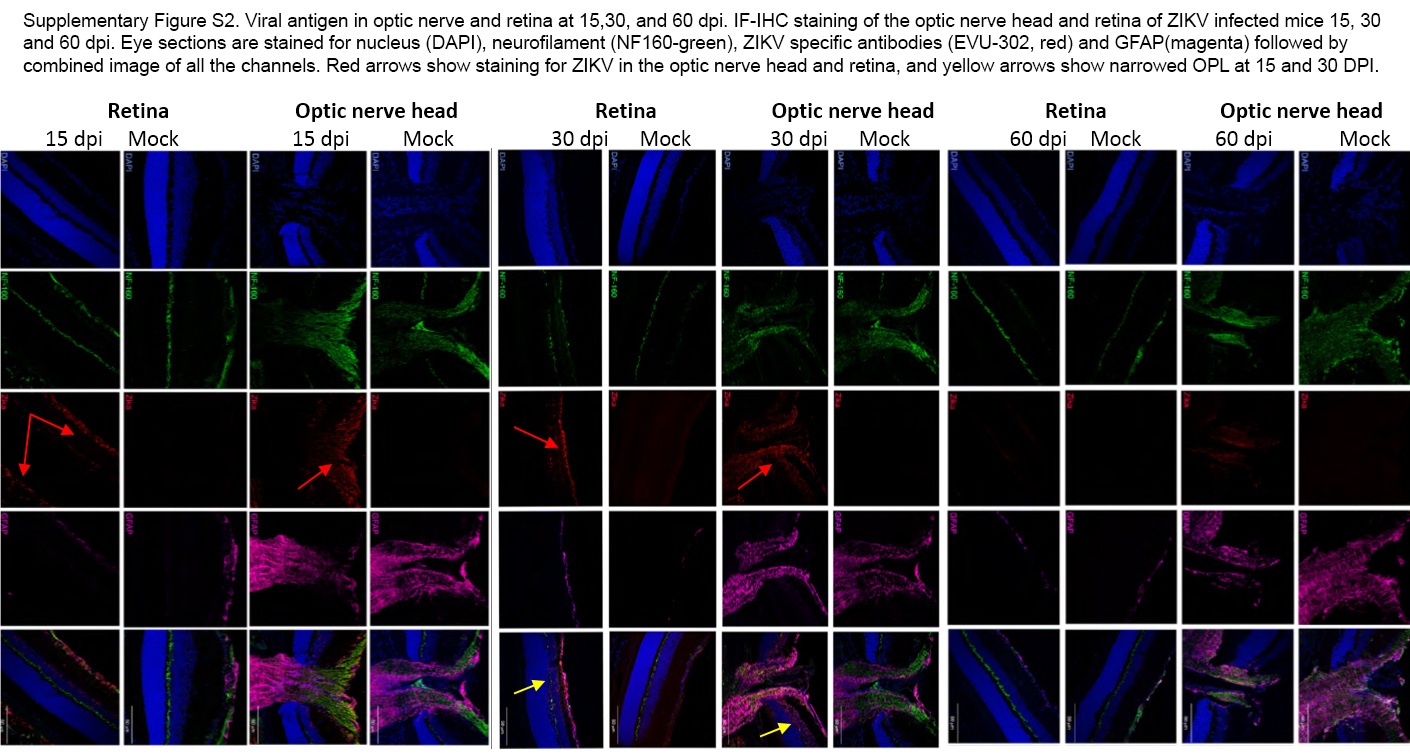

Supplement: Supplementary file 2 — Supplementary Figure S2 [file 41426_2018_96_MOESM2_ESM.jpg]

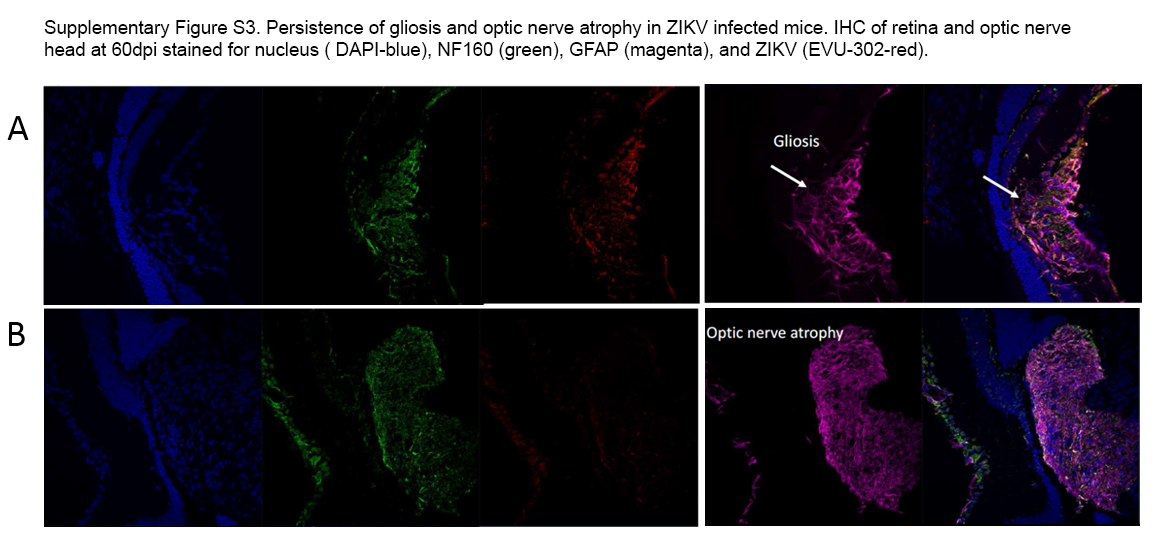

Supplement: Supplementary file 3 — Supplementary Figure S3 [file 41426_2018_96_MOESM3_ESM.jpg]

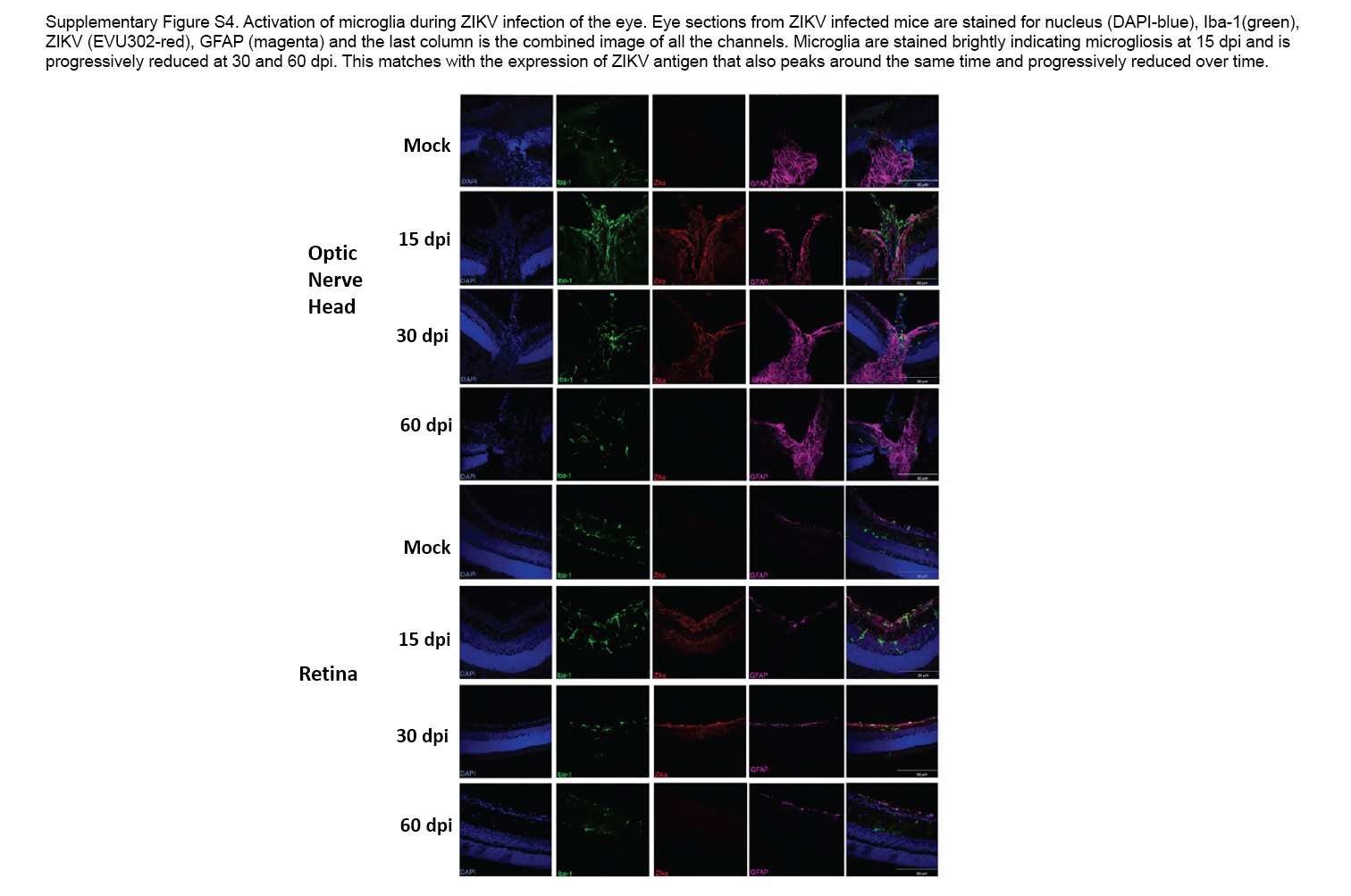

Supplement: Supplementary file 4 — Supplemental Figure S 4 [file 41426_2018_96_MOESM4_ESM.jpg]

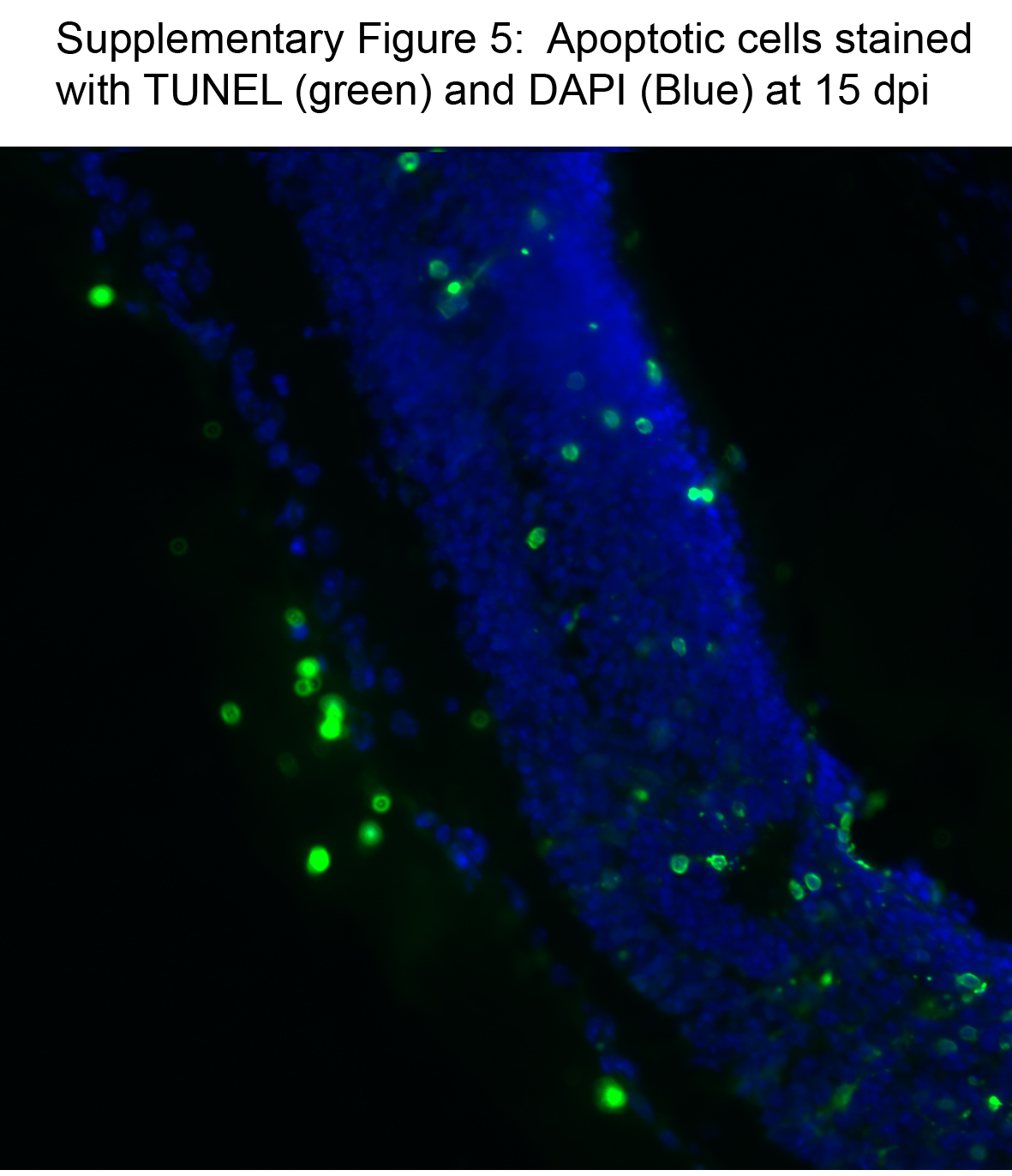

Supplement: Supplementary file 5 — Supplementary Figure S5 [file 41426_2018_96_MOESM5_ESM.jpg]
